# The Thermal Sensitivity Test in Evaluating Outcome after Peripheral Nerve Injury

**DOI:** 10.1155/2015/528356

**Published:** 2015-06-23

**Authors:** Marcin Ceynowa, Tomasz Mazurek, Rafał Pankowski, Marek Rocławski, Mariusz Treder

**Affiliations:** Department of Orthopedic Surgery, Medical University of Gdańsk, Ulica Nowe Ogrody 1-6, 80-803 Gdańsk, Poland

## Abstract

The purpose of this study was to evaluate the ability to discriminate temperatures in patients following peripheral nerve injury. Knowing that temperature sensibility is mediated by different receptors, the scores were compared to other functional hand scores in order to determine whether the ability to discriminate temperatures is restored to a different extent compared with other commonly evaluated hand function modalities. The test was performed using the NTE-2 device (Physitemp Instruments Inc., 154 Huron Avenue, Clifton, New Jersey, USA). Out of 57 patients, 27 had normal thermal discrimination scores, and 9 could not tell the temperatures apart in the differences set on the measuring device. Overall, patients with better thermal discrimination had also better hand function as evaluated with different methods. However, some patients who did regain the ability to differentiate temperatures correctly did not have any measurable return of hand function in other tests. Thermal discrimination scores correlated similarly with different functional scores, except for vibration sensibility, which did not show any significant correlation. The development and severity of cold intolerance seem to be unrelated to temperature sense.

## 1. Introduction

Regeneration after peripheral nerve injury has been extensively studied. Many studies and authors have contributed to our better understanding of hand function after nerve injury and regeneration. The evaluation of nerve function is performed with many different methods that assess different sensory modalities and the contribution of these modalities to overall hand function. However, to the best of the author's knowledge, the ability of discriminating between different temperatures following peripheral nerve injuries has not been evaluated before [1–10].

The sensory function is a complex modality that requires correct functioning of several different receptors, nerve fibers, nervous system pathways, and brain centers. There are several kinds of receptors that respond to different stimuli. Transection of a peripheral nerve breaks the information flow from skin and internal organ receptors to specialized brain centers [11]. Commonly used evaluation methods, such as Medical Research Council sensory evaluation test, the 2-point discrimination test, or Semmes-Weinstein monofilament test, are based on simple touch sensibility or are based on both the touch sensibility and the sensory gnosis, such as the Rosén and Lundborg test or Moberg pick-up test. Some patients after peripheral nerve injury are not able to recognize the shape and texture of an object or the extent of pressure applied to the affected hand. Regaining protective touch sense, the ability to feel the limb and to recognize the fact that it is touching an object, helps protecting the limb from additional injury and allows some hand function. It seems reasonable to think that the ability to discriminate between warm and cold objects is an important factor in this protective touch [5, 12].

The thermal discrimination test, that is, measuring the ability to differentiate temperature levels, is used in evaluating diabetic neuropathy or vibration induced neuropathy. This evaluation is performed using different methods. The measurement is usually based on determining the temperature level which elicits pain during warming or cooling of the extremity [13–15].

The purpose of this study was to evaluate the ability to discriminate temperatures in patients following peripheral nerve injury. Knowing that temperature sensibility is mediated by different receptors, the scores were compared to other functional hand scores in order to determine whether temperature sensibility is restored to a different extent compared with other commonly evaluated hand function modalities.

## 2. Material and Methods

The study was designed as a retrospective cohort study. All patients who sustained a confirmed intraoperatively complete nerve laceration (neurotmesis according to Seddon) at the forearm level were asked for a follow-up examination. The patients were operated on in our department between 2004 and 2010 (78 patients). Patients with other types of nerve injuries as well as known diseases that could influence nerve function, such as diabetes, alcohol abuse, or neurological disorders, were not included in the study. There were 57 patients enrolled for the study after median or ulnar nerve injury (response rate is 73%). The surgical procedures were performed by orthopedic trauma surgeons on emergency duty who performed primary repair and 4 hand surgeons who performed secondary repair. The mean follow-up period was 4 years from the surgical procedure (range: 17 months to 8 years).

The examination was performed in the Clinical Physiology Department, Medical University of Gdansk, after patients adjusted to room temperature to avoid interference of cold intolerance symptoms of the examination. Every test in the examination was performed by a single rater.

Primary epineural repair was performed in 31 patients, from which 18 had median nerve and 13 had ulnar nerve repair. Primary repair was performed within 24 hours from injury. Mean age at the time of the initial injury was 35 years (range 16–56; SD 13,1; Me 35), predominantly male patients (28 men and 3 women). The dominant extremity was injured in 14 patients and the nondominant one in 17 patients. All patients were in overall good health condition; 5 of them had mild hypertension, 12 patients were regular cigarette smokers, and 1 patient had asthma.

A secondary repair was performed in overall 26 patients, from which 12 had median nerve and 14 had the ulnar nerve repaired. Mean age at the time of the initial injury was 34,7 years (range 16–64; SD 14,3; Me 29), predominantly male patients (28 men and 3 women). The dominant extremity was injured in 13 patients and the nondominant one in 13 patients. All patients were in overall good health condition; 3 of them had mild hypertension, 8 patients were regular cigarette smokers, and 1 patient had gout.

The reason for secondary repair was a missed initial diagnosis in 11 patients and a failed primary repair with formation of a neuroma in 15 patients. Neurorrhaphy was performed in 6 patients. Interfascicular nerve grafting was performed in 20 patients, whenever the nerve defect after excision of a neuroma did not allow direct nerve suture without tension. Average time from injury to repair in patients with missed diagnosis was 4,6 months (range: 3 to 6 months) and in patients with a failed primary repair 8,6 months (range: 6 to 13 months). Average length of the defect was 3,8 cm (range: 3 to 6,5 cm). The source of the graft was the sural nerve. All patients reported slight numbness on the dorsum of the foot from the skin area supplied by the nerve, which they considered irrelevant. Three of those patients reported moderate cold intolerance in the foot, however, which they considered of little relevance not affecting their everyday activities.

Testing methods included the thermal discrimination test, the protocol described by Rosén and Lundborg, the DASH questionnaire, and sensory vibration evaluation using Vibratron II device (Physitemp Instruments Inc., 154 Huron Avenue, Clifton, New Jersey, USA). All outcome measures were validated in literature [3, 4, 6, 7, 10, 13, 14].

Sensibility was tested on the pulp of the index finger in the median nerve-injured hands and small finger in the ulnar nerve-injured hands. The contralateral finger serves as control. Motor function, cold intolerance, and hypersensitivity were evaluated according to the Rosén and Lundborg assessment score described below.

The temperature measurement test was performed using the NTE-2 device (Physitemp Instruments Inc., 154 Huron Avenue, Clifton, New Jersey, USA). It consists of two metal testing plates; the temperature of one is fixed and the other's temperature changes during testing. The test was performed after the patients adapted to the room temperature, that is, approximately 22°C. The patient was instructed to place the tested finger on one of the metal testing plates for approximately 1 second and then move the finger to the second plate to tell which plate was cooler.

The temperature differences are set by the manufacturer as standard deviations from normal, established in different age groups. There are 6 possible levels of function for each age group (Table 1); in this study, since only one patient scored 1, patients who scored 0 and 1 were included in group 1. One of the plates serves as control, with its temperature set for 25°C, and the temperature of the other is set to be cooler or warmer. The test is begun with temperature difference of 5 SD (standard deviation) between the two plates. If the patient recognizes the cooler plate correctly the temperature difference is lowered one level down; if the answer is incorrect the difference in plate temperatures is increased one level up. The level of temperature differentiation is established as the smallest plate temperature difference recognized correctly. The test is performed three times to exclude the element of chance, with at least two corresponding answers considered to be the final test result. If all three answer series were different the patient was supposed to be excluded from the evaluation. This, however, has not happened in our study. The whole test was performed twice, in the beginning and in the end of the evaluation, with approximately 1-hour time interval between them [14].

The results were compared to the results obtained from the Rosén-Lundborg protocol and its cold intolerance score as well as separately with the Semmes-Weinstein monofilament test, the Dellon static 2-point discrimination test, the Medical Research Council power grading score [1, 7], and the vibration sensibility score [14, 15]. The patients filled in the Disabilities of the Arm, Shoulder, and Hand (DASH) questionnaire score [16, 17], in a validated Polish translation. We correlated different assessment scores with the DASH questionnaire to see whether temperature perception influences patient's subjective opinion on hand function to a greater or lower extent than traditionally evaluated sensory or motor function.

The details of the Rosén and Lundborg assessment score are given in Table 2. It is composed of 3 domains: sensory, motor, and pain/discomfort. The sensory domain is comprised of the following tests: the Semmes-Weinstein monofilament test, the static 2-point discrimination test, the shape/texture identification test (STI), and the Sollerman hand function test (tasks 4, 8, and 10) [3, 5, 6].

The motor domain is comprised of the Medical Research Council power grading for nerve-specific muscles and grip strength measured with the Jamar hydraulic hand dynamometer.

The cold intolerance and pain/hypersensitivity score is in fact a self-assessment questionnaire of symptoms perceived by the patient. The patient is asked to assess his/her own symptoms in a four-grade descriptive scale. Symptoms range from “none/mild” to “hindering function” when pain, stiffness, and excessive feeling of cooling in the hand do not allow them to perform even simplest tasks with the injured hand, at a temperature where the other uninjured hand is painless and maintains normal function.

A brief comparison of results between primary and secondary repair in median and ulnar nerve was performed to allow comparison with other studies on the results of nerve repair in adults.

The study was approved by the Independent Bioethical Committee for Scientific Research at the Medical University of Gdansk, Poland.

Statistics was performed using SPSS for Windows v.16.0. The Pearson correlation coefficient was used to determine correlations between evaluated factors. Correlations were considered significant with *p* < 0.05.

## 3. Results

Based on the test results, the patients were divided into 5 groups, given their ability to differentiate temperatures. As seen in Table 3 all groups were similar according to age, type of nerve injured, and mode of repair.

Out of 57 patients, 27 had normal temperature differentiation scores and 9 could not tell the temperatures apart in the differences set on the measuring device.

Cold intolerance as evaluated according to Rosén and Lundborg score was reported in overall 34 patients (59,6%), with 4 patients who had moderate symptoms (7%), 13 (22,8%) who described their symptoms as disturbing, and 17 (29,8%) who had symptoms severe enough to hinder hand function.

**Figure 1:**
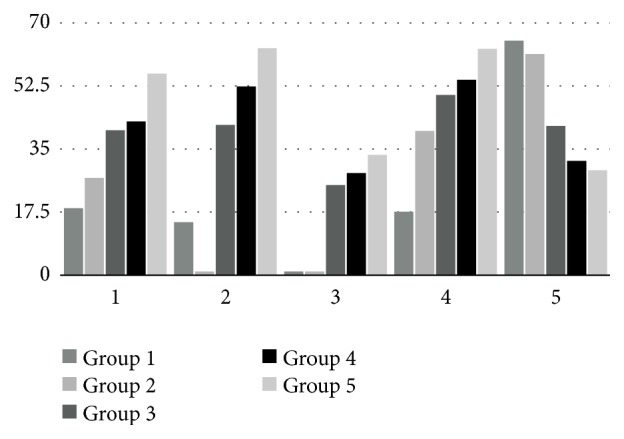
Mean functional scores in groups according to temperature scores. 1: mean Rosén-Lundborg score. 2: mean cold intolerance score. 3: mean static 2-point discrimination test. 4: mean Semmes-Weinstein monofilament test score. 5: mean DASH score (the lower the score, the better the result) (all scores are given in percentages for clarity).

Overall, patients with better thermal sensitivity scores had better results in other functional scores (Figure 1). Some patients who did regain the ability to differentiate temperatures correctly did not have any measurable return of hand function in other tests (Table 4). The thermal sensitivity test scores correlated positively with other functional scores, indicating that patients with poor thermal sensitivity scores scored also poor in other functional scores (Table 5). As seen from Table 5, temperature scores correlated similarly with different functional scores, except for vibration sensibility, which did not show any significant correlation. The correlations of the DASH questionnaire and hand function tests are shown in Table 6. To allow comparisons with other studies, the results of primary versus secondary repair are given in Table 7.

## 4. Discussion

The ability to feel temperatures and temperature differences between objects is an important feature in sensory function of the human body. It helps to protect from thermal injury, both heat and cold, and contributes to recognition of the shape and texture by assessing temperature differences between objects and the surfaces [11].

Peripheral nerve injury disconnects all sensory receptors from sensory brain centers. Nerve regeneration allows reinnervation of those receptors to various degrees. However, restoration of simple touch sensibility does not necessarily mean restoration of tactile gnosis and in consequence of full hand function. Correct hand function requires restoration of a number of other modalities, such as correct discrimination of pain stimuli, sudomotor and vasomotor function, and normal tissue trophic. Their dysregulation may result in pain hypersensitivity, cold intolerance, skin trophic changes, or complex regional pain syndrome [1, 18, 19].

Most commonly used assessment methods evaluate only mechanoreceptors, such as Meissner, Pacinian corpuscles, and Ruffini endings, together with motor and sometimes sympathetic nerve function, leaving thermoreceptors aside. Subjective hand function questionnaires such as the DASH score or the cold intolerance/pain domain in the Rosén and Lundborg evaluation score is aimed at filling the gap between simple clinical evaluation of basic motor and sensory recovery and the complexity of patient's complaints after peripheral nerve injury [1, 11, 17].

In this study, we evaluated the thermoreceptors and compared the degree of their restoration of function to other recognized evaluation methods. The goal was to determine whether the sensibility to thermal stimuli differs from the sensibility to mechanical stimuli. The results show that the thermal sensibility recovers to different extent in different patients (Table 4). In patients who achieved poor thermal sensibility scores there are a higher percentage of patients with poor mechanosensory function (as evaluated in static 2-point discrimination test and Semmes-Weinstein monofilament test) and conversely there are a higher percentage of patients with better mechanosensory function in groups with better thermosensory functions (Table 4).

In our study approximately half of the patients regained full thermal discrimination, whereas only 10 (17%) had normal 2-point discrimination and only 4 (7%) had normal touch sensibility as evaluated in Semmes-Weinstein monofilament test. It seems that correct thermal discrimination returns to a greater extent than sensibility to mechanical stimuli. Novak et al. [20] found that perception of pain and temperature is the first to return. Novak et al. suggested that quicker return of thermal perception is due to the fact that it is mediated by small nerve fibers which regenerate faster. However, this does not necessarily mean that small nerve fibers regenerate to a greater extent. A reasonable different explanation would be that the thermal stimuli are perceived by free nerve endings [20], which will maintain their function without a specialized receptor, such as a mechanoreceptor for touch sensibility that an ingrowing nerve fiber has to reach to receive appropriate stimuli.

The correlation with other hand function domains both sensory and motor is significant with most of them (Table 5). However, this can be explained by the fact that in a regenerating nerve all nerve fibers and consequently all evaluated hand function modalities regenerate to some extent. Since different nerve fibers and receptors are responsible for different evaluated modalities, this can be explained by the fact that these nerve fibers regenerate independently from each other, but along the same anatomical path, that is, the nerve trunk, which when restored provides the same support to all fibers. The differences in function should be attributed to different regeneration potential of various types of nerve fibers. The other reason is the response to denervation of target organs, where those fibers that require specialized receptors (e.g., mechanoreceptors) or effectors (muscle fiber) tend to have a comparably worse function than those that need only free nerve endings to receive stimuli [1, 11, 20].

A correlation that is worth pointing out is the one with pain hypersensitivity (see Tables 4 and 5). Pain, similarly to temperature, is also mediated with small fibers, with free nerve endings as receptors [2, 3, 11]. Absence of pain hypersensitivity was found in 25 patients, percentage similar to this of normal temperature differentiation. That would suggest that similar types of nerve fibers and receptors would regain correct function to a similar extent in the same patient. However, not every patient with normal temperature differentiation had normal sensitivity to pain, and vice versa (Table 4). The correlation between those modalities was not much different from correlations of temperature scores with other evaluation scores (Table 5). The statement that nerve fibers which are responsible for different hand function modalities regenerate independently from one another seems to be valid in this case also. It must be noted that absence of pain hypersensitivity does not necessarily mean normal pain perception or normal pain fiber function. However, to the best of our knowledge, any widely accepted method of assessment of pain perception has not yet been developed [2, 3].

Given the limitations of clinical evaluation of hand function, subjective means of assessing hand function were developed, such as the DASH questionnaire for upper extremity dysfunction. It involves some activities of daily living as well as some complaints a patient might have regarding his/her hand function. The DASH questionnaire was correlated with different assessment scores, including temperature score, to see whether temperature perception influences patient's subjective opinion on hand function to a greater or lower extent than traditionally evaluated sensory or motor function [16, 17].

As seen from Table 6, highest correlation of subjective DASH score was found with the Rosén-Lundborg score, probably a most complete hand evaluation test in use nowadays. Temperature score correlates highly with DASH. However, if we consider a patient's good performance in the activities of daily living to be our goal of treatment, there are other scores that are much simpler and quicker to perform, which would predict a hand's usefulness in everyday life more accurately. It seems that for everyday life touch sensibility the absence of hypersensitivity to pain and absence of cold intolerance are more important factors that influence hand function than the ability to differentiate temperatures.

The presence of cold intolerance proves to be a very important factor that negatively influences hand function, both in this study and in a number of other studies [19, 21]. It is classically described as “an icy cold feeling that can progress to pain” or “an exaggerated or abnormal reaction to cold exposure of the injured part causing discomfort or the avoidance of cold” [22, 23]. In this study we tried to find a connection between the ability to discriminate temperatures and the occurrence and severity of cold intolerance symptoms.

The occurrence of cold intolerance is difficult to predict and when present it tends to persist for years. Its pathophysiology is somewhat unclear but it is generally accepted that autonomic dysfunction is responsible for its development [23]. The prevalence of cold intolerance in our study corresponded to the literature [23–25].

The temperature sense would influence cold intolerance in two possible ways. An abnormal feeling of cold could be caused by hyperactive thermal nerve fibers that are causing patient's discomfort in temperatures normally not perceived as too cool or an abnormal activity of thermal fibers could trigger pathological response to cold based on a reflex arc with the autonomic system.

Cold intolerance correlates average with thermal sensitivity scores (Table 5), similarly to other hand function modalities both sensory as well as the MRC muscle power grading, which are all mediated by other nerve fibers and/or receptors. This fact does not allow presuming the presence of any special nerve pathway between the temperature pathway and the autonomic system, responsible for the development and severity of cold intolerance. Moreover, inability to differentiate temperatures does not exclude the presence of cold intolerance symptoms, or conversely, cold intolerance may develop in patients with normal temperature sense (Table 4). Cold intolerance seems to be unrelated to temperature sense.

The thermal sensitivity test requires further investigation. The ability to discriminate temperatures has not been investigated extensively. The limitation of the study is that it is a retrospective cohort study. A prospective study assessing gradual changes of the regeneration of temperature sensitivity would perhaps give interesting insights into cold intolerance studies, as well as our knowledge on the outcome after peripheral nerve injuries. A brief comparison with other studies shows that results of primary repair in this study differ to some extent from those obtained by other authors, showing inferior results of sensory recovery in our study when compared to other studies; however, the results of secondary repair are comparable with the results of other authors [9, 26–28]. A detailed comparison of the results requires further investigation that takes into account other factors that may have influenced outcome (age, scale of the injury, among other) and is not within the scope of this paper.

## 5. Conclusions

1. Correct temperature differentiation returns in approximately half of the patients with injuries to the median and ulnar nerves. It correlates with better overall hand function, as evaluated in other hand function tests; however, good temperature sensibility can be seen in patients with poor touch sensibility.
2. Good temperature sensibility can contribute to hand protection sensibility; however, it is probably less important than touch sensibility.
3. Temperature sensibility is unrelated to the development and severity of cold intolerance.

## Figures and Tables

**Table 1:** Scores for temperature measurement test with NTE-2.

	Group 5	Group 4	Group 3	Group 2	Group 1	
Age	Normal	Borderline normal	Mild neuropathy	Mild moderate neuropathy	Severe moderate neuropathy	Severe neuropathy
	(Correct at 2,5 SD)	(Correct at 3,5 SD)	(Correct at 5 SD)	(Correct at 6,5 SD)	(Correct at 8 SD)	(Incorrect at 8 SD)
<30	1,4	1,7	2,1	2,6	3	>3,0
31–45	1,4	1,8	2,2	2,7	3,2	>3,2
46–60	1,6	1,9	2,5	3	3,5	>3,5
>60	1,9	2,3	2,9	3,6	4,2	>4,2

Values in the table are the lowest correctly recognized temperature difference. Temperature differences are presented in degrees Celsius.

**Table 2:** The Rosén and Lundborg assessment score.

	Score	Testing area	Result
Sensory domain			
Semmes-Weinstein monofilament test	0 = not testable 1 = filament 6.65 2 = filament 4.65 3 = filament 4.31 4 = filament 3.61 5 = filament 2.83	Median nerve: (i) pulp of finger II, (ii) base of finger II, (iii) pulp of the thumb Ulnar nerve: (i) pulp of finger V, (ii) base of finger V, (iii) hypothenar area	0 to 5 for each tested area; the result is the sum of each test Result: 0–15
Static 2-point discrimination test	0 = >15 mm 1 = 11–15 mm 2 = 6–10 mm 3 = <6 mm	Median nerve: pulp of finger II Ulnar nerve: pulp of finger V	0 to 3 for each nerve Result: 0–3
Shape/texture identification test	0 = not testable Scores 1 to 3 = correct recognition of shapes/textures of different sizes	Median nerve: pulp of finger II Ulnar nerve: pulp of finger V	0 to 6 for each nerve Result: 0–6
Sollerman test	1: doing up 4 buttons of different sizes 2: putting 4 nuts on bolts of different sizes 3: picking 4 coins of different sizes from a purse	Task performed single-handed	0–4 for each test Result: 0–12

Motor domain			
Medical Research Council power grading	0: no muscle contraction 1: visible muscle contraction but no joint movement 2: active joint movement with gravity eliminated 3: movement against gravity 4: movement against gravity and some resistance 5: full power against resistance	Median nerve: palmar abduction of the thumb Ulnar nerve: (i) abduction of fingers II and V (ii) adduction of finger V	Median nerve: 0–5 Ulnar nerve: 0–15
Grip strength	Mean of 3 trials (evaluated with Jamar dynamometer)	Grip strength of both hands	Grip strength of the uninjured hand is considered normal

Pain/discomfort			
Cold intolerance Pain/hypersensitivity	0: hindering function 1: disturbing 2: moderate 3: none/mild	Patient's own estimation of the problem	Result: 0–3

Each domain is scored as follows: sum of the results for the examined nerve for the domain is divided by the expected normal result for the domain, for example, sensory domain: sum of scores/(15 + 3 + 6 + 12).

The result from every domain is in fact a percentage. Maximal score for each domain is 1; maximal score for the whole assessment is 3.

**Table 3:** Patients according to temperature scores-group characteristics.

Groups according to temperature score	Nerve	Type of repair	Mean age	Gender
1 (*n* = 9)	Median: 3 Ulnar: 6	Primary repair: 4 Secondary neurorrhaphy: 1 Nerve grafting: 4	36,5 (range: 24–62)	M: 8 F: 1

2 (*n* = 2)	Median: 1 Ulnar: 1	Primary repair: 1 Secondary neurorrhaphy: 0 Nerve grafting: 1	41 (range: 39–43)	M: 2 F: 0

3 (*n* = 12)	Median: 7 Ulnar: 5	Primary repair: 6 Secondary neurorrhaphy: 2 Nerve grafting: 4	39,7 (range: 21–56)	M: 11 F: 1

4 (*n* = 7)	Median: 4 Ulnar: 3	Primary repair: 4 Secondary neurorrhaphy: 2 Nerve grafting: 1	29 (range: 16–50)	M: 6 F: 1

5 (*n* = 27)	Median: 15 Ulnar: 12	Primary repair: 16 Secondary neurorrhaphy: 1 Nerve grafting: 10	32 (range: 16–64)	M: 24 F: 3

**Table 4:** Number of patients with according hand function score in given temperature score group.

Groups according to thermal discrimination score	Pain hypersensitivity	Cold intolerance score	Static 2-point discrimination test score	Semmes-Weinstein monofilament test score
1 (*n* = 9)	**Hindering** ** function**:** 4** **Disturbing**:** 1** Moderate (*none*) **None/minor**:** 4**	**Hindering function**:** 7** Disturbing (*none*) **Moderate: 2** None/minor (*none*)	**>15 mm**:** 9** **11**–**15 mm (*none*)** **6**–**10 mm (*none*)** **<6 mm (*none*)**	**0**:** 5** **1**:** 2** **2**:** 1** 3 (*none*) **4**:** 1** 5 (*none*)

2 (*n* = 2)	Hindering function: 1 Disturbing (*none*) Moderate: 1 None/minor (*none*)	Hindering function: 2 Disturbing (*none*) Moderate (*none*) None/minor (*none*)	>15 mm: 2 11–15 mm (*none*) 6–10 mm (*none*) <6 mm (*none*)	0: 1 1 (*none*) 2 (*none*) 3 (*none*) 4: 1 5 (*none*)

3 (*n* = 12)	Hindering function: 2 Disturbing: 2 Moderate: 2 None/minor: 6	Hindering function: 5 Disturbing: 2 Moderate: 2 None/minor: 3	>15 mm: 8 11–15 mm: 1 6–10 mm: 1 <6 mm: 2	0: 1 1: 2 2: 2 3: 5 4: 1 5: 1

4 (*n* = 7)	Hindering function: 2 Disturbing: 1 Moderate: 2 None/minor: 2	Hindering function: 3 Disturbing (*none*) Moderate: 1 None/minor: 3	>15 mm: 4 11–15 mm: 1 6–10 mm: 1 <6 mm: 1	0: 1 1 (*none*) 2: 2 3: 2 4: 1 5: 1

5 (*n* = 27)	**Hindering function**:** 1** **Disturbing**:** 6** **Moderate**:** 6** **None/minor**:** 14**	**Hindering function**:** 6** **Disturbing**:** 2** **Moderate**:** 8** **None/minor**:** 11**	**>15 mm**:** 14** **11**–**15 mm**:** 6** 6–10 mm (*none*) **<6 mm: 7**	**0**:** 1** **1**:** 2** **2**:** 4** **3**:** 9** **4**:** 7** **5**:** 4**

The table shows the number of patients who achieved a given functional score (pain/hypersensitivity, cold intolerance, static 2-point discrimination score, and Semmes-Weinstein monofilament score) in each group of patients who achieved a given thermal discrimination score. The percentage of patients with better functional scores is higher in groups of patients with better thermal discrimination scores. The differences are best seen between groups 1 (no ability to differentiate temperatures) and 5 (normal thermal sensitivity score).

**Table 5:** Correlations of temperature scores with other hand function evaluation scores.

Evaluation method	Correlation with temperature scores	Significance
Rosén-Lundborg evaluation test	0,56	*p* < 0,001
Sensory domain in Rosén-Lundborg evaluation test	0,49	*p* < 0,001
Cold intolerance score in Rosén-Lundborg evaluation test	0,44	*p* < 0,001
Semmes-Weinstein monofilament test	0,54	*p* < 0,001
Static 2-point discrimination test	0,36	*p* = 0,01
Vibration sensibility	0,13	*p* = 0,34
Medical Research Council power grading	0,42	*p* < 0,001
DASH questionnaire	−0,49	*p* < 0,001
Pain hypersensitivity	0,52	*p* < 0,001

**Table 6:** Correlation of subjective and objective means of hand function evaluation.

Evaluation method	Correlation with DASH	Significance
Rosén-Lundborg evaluation test	−0,76	*p* < 0,001
Sensory domain in Rosén-Lundborg evaluation test	−0,38	*p* < 0,001
Cold intolerance score in Rosén-Lundborg evaluation test	−0,73	*p* < 0,001
Semmes-Weinstein monofilament test	−0,63	*p* < 0,001
Static 2-point discrimination test	−0,43	*p* < 0,001
Vibration sensibility	−0,39	*p* < 0,05
Medical Research Council power grading	−0,46	*p* < 0,001
Temperature discrimination	−0,49	*p* < 0,001
Pain hypersensitivity	0,61	*p* < 0,001

**Table 7:** Comparison between primary and secondary repairs.

	Static 2-point discrimination test	MRC power grading	Rosén and Lundborg
Primary repair median nerve (*n* = 18)	>16 mm = 10 11–15 mm = 2 6–10 mm = 2 <6 mm = 4	M0–M2 = 2 M3 = 2 M4 = 2 M5 = 10	Mean: 1.61 (53.69%) SD: 0.72

Primary repair ulnar nerve (*n* = 13)	>16 mm = 7 11–15 mm = 2 6–10 mm = 1 <6 mm = 3	M0–M2 = 9 M3 = 1 M4 = 2 M5 = 1	Mean: 1.3 (43.38%) SD: 0.78

Secondary repair median nerve (*n* = 12)	>16 mm = 8 11–15 mm = 1 6–10 mm = 1 <6 mm = 2	M0–M2 = 1 M3 = 2 M4 = 0 M5 = 8	Mean: 1.08 (36.17%) SD: 0.77

Secondary repair ulnar nerve (*n* = 14)	>16 mm = 9 11–15 mm = 3 6–10 mm = 1 <6 mm = 1	M0–M2 = 10 M3 = 2 M4 = 2 M5 = 0	Mean: 1,17 (29.04%) SD: 0.51

The table shows the number of patients with a given functional score. The Rosén and Lundborg total score is given in original scores 0–3 with a percentage of the total score given in brackets.
